# Clinical Diagnosis and Management of Gradual Onset Cannabis-Induced Psychosis Following the Consumption of Delta-8-Tetrahydrocannabinol

**DOI:** 10.7759/cureus.55464

**Published:** 2024-03-03

**Authors:** Edward E Denton, Sophia S Jung, Christian Angelo I Ventura

**Affiliations:** 1 Department of Emergency Medicine, University of Arkansas for Medical Sciences, Little Rock, USA; 2 Department of Health, Behavior, and Society, Johns Hopkins Bloomberg School of Public Health, Baltimore, USA

**Keywords:** substance use disorder, hemp, marijuana, delta-8, psychosis, cannabis

## Abstract

Cannabis-induced psychosis (CIP) is an increasingly acknowledged psychiatric phenomenon observed in vulnerable patients exposed to cannabis. This brief case report details a male patient in his 20s, who presents to the ED with derealization two days after ingesting a gummy worm containing delta-8-tetrahydrocannabinol (Δ8-THC). Two days post-ED discharge, the patient gradually developed symptoms of religious-themed psychosis and was prescribed 10 mg of aripiprazole daily. The patient seemingly recovered within four days of starting treatment. This paper contributes to the limited literature pertaining to CIP and discusses implications for diagnostics and treatment in the ED setting.

## Introduction

While cannabis is recognized for its therapeutic potential in managing certain medical conditions, its non-medical utilization has raised alarms over mental health disorders, cognitive impairments, and a variety of physical health issues [1]. The evolving legal and societal landscape surrounding cannabis has notably led to an increase in its consumption among young adults in their 20s, a group often navigating significant life changes and susceptible to peer influences [2]. The passage of the Agriculture Improvement Act of 2018 marked a turning point, federally legalizing the sale and transportation of products containing hemp-derived cannabinoids with less than 0.3% tetrahydrocannabinol (THC), thus catalyzing the hemp industry's growth in the United States. However, the burgeoning presence of hemp-derived synthetic cannabinoids such as delta-8-tetrahydrocannabinol (Δ8-THC), produced through a synthetic process in the lab from cannabidiol (CBD) found in hemp, may warrant further study. This synthesis typically involves a cyclization reaction that transforms CBD into Δ8-THC, a chemical cousin of Δ9-THC but with a slightly altered molecular structure featuring a double bond isomer. This small chemical modification makes Δ8-THC a partial agonist of the CB1 receptor, similar to Δ9-THC, yet its effects and potency can differ [3].

Initial studies suggest that Δ8-THC might still provoke cognitive effects such as difficulty in thinking and time distortion in adults, alongside euphoria and agitation in children, raising concerns about its safety profile [4]. Beyond Δ8-THC, the market has seen the emergence of other synthetically derived cannabinoids such as Δ10-THC, THC-P, THC-JD, and HHC. These compounds, while chemically distinct from Δ9-THC, can produce psychoactive effects akin to it when consumed in substantial amounts. This similarity to the psychoactive effects of Δ9-THC underscores the potential for misuse and health risks, especially among young populations prone to experimentation [5].

The legal gap that allows the shipment of these hemp-derived products across state lines, unlike Δ9-THC, lies in their derivation from hemp and their THC content being below the 0.3% threshold. This legal distinction, however, does not fully address the potential for misuse and health risks associated with these compounds. The lack of stringent regulations on the quality and safety of these products further complicates the public health perspective, leading to a scenario where consumers might be exposed to untested and potentially harmful substances [6,7]. The recent imposition of sales restrictions by several states reflects growing apprehension toward these synthetic cannabinoids, echoing the concerns traditionally associated with illicit cannabis use but compounded by the uncertainties surrounding the quality and safety of these novel products.

The escalating prevalence of cannabis utilization has led to heightened awareness of the potential detrimental consequences linked to its consumption. Although most cannabis users do not exhibit severe psychiatric manifestations, a specific subset of individuals with predisposing elements, such as personal or familial psychiatric disorder history, may face an elevated risk of cannabis-induced psychosis (CIP) diagnosis. This condition necessitates further exploration to enhance comprehension, diagnostic accuracy, and clinical management.

CIP is characterized by various symptoms, including hallucinations, delusions, disordered thinking patterns, and changes in behavior, which generally manifest during or soon after cannabis consumption. The precise biological mechanisms underlying CIP are not yet fully understood; nevertheless, it is theorized that a mix of individual genetic and environmental aspects, in conjunction with the pharmacological characteristics of cannabis constituents, could play a role in the development of psychotic symptoms [8]. Identifying CIP can be particularly challenging, as the clinical manifestations can mimic those of other psychiatric disorders, requiring the elimination of alternative diagnoses and the consideration of potential substance interactions before arriving at a conclusive diagnosis.

At present, the relationship between these other hemp-derived compounds and CIP has not been widely explored; however, contemporary findings suggest that CIP can similarly be present with the consumption of these other hemp-derived compounds such as Δ8-THC [9]. The use of cannabis is associated with the early onset of psychosis; however, the underlying mechanisms and risk factors are unknown and heavily debated. Patients with psychosis and a history of cannabis use have been found to have symptom onset three years earlier compared to psychosis patients with no history of use [10]. However, other factors such as sex and age need to be examined further to clearly understand the risk relationship between cannabis and psychosis.

Successful treatment of CIP is crucial to prevent additional complications and promote recovery. Typically, therapeutic strategies encompass a blend of pharmacological and non-pharmacological interventions, with antipsychotic medications serving as the foundation for drug therapy [11]. Treatment of underlying cannabis use disorder may also be clinically necessary. In addition to the use of antipsychotic drugs, delivering supportive care and imparting psycho-education to the patient and their family members is essential in expediting recovery and reducing the likelihood of recurrence.

## Case presentation

A male patient in his 20s presented to the emergency department (ED) with a chief complaint of derealization and worsening altered mental status. The patient’s spouse reported the patient to be an unreliable historian and provided much of the clinical history and context. There was no family history of psychosis or mood disorder; however, the patient's mother has previously been hospitalized for suicidal ideation and an eating disorder in her youth.

Approximately four days prior to ED arrival, the patient had an inadvertent ingestion of a sour gummy worm that was allegedly labeled to contain “Delta” according to the packaging, which a friend had ordered via mail. This packaging was identified as containing psychoactive legalized hemp-derived cannabinoids while remaining under the required limits of Δ9-THC. Two hours post-consumption, the patient experienced intoxicating-like effects for approximately 30 minutes, which ultimately restored. Two days after initial consumption, the patient’s spouse reported that the patient began to exhibit noticeable signs of atypical behavior, which gradually progressed. Initially, the patient began to forget his wedding ring and phone when leaving the house, which he did not realize until hours later. He also began to miss work and school assignments, which were unusual. The patient’s spouse decided to bring the patient to the ED, when he witnessed the patient run two red traffic lights while driving and was ultimately confused about the destination.

The patient’s height was 5’10” and weight was 180 lbs, and he was a white male with a history of generalized anxiety disorder, excoriation disorder, and non-alcoholic fatty liver disease (NAFLD). He takes 100 mg sertraline daily, which he has been prescribed for at least four years without issue. At the time of triage, he was afebrile and normotensive with all unremarkable vital signs. He denies suicidal or homicidal ideation. Upon examination, he was alert and oriented to time, place, location, and identity, although there was a noticeable five- to six-second delay in responding to questions, which his spouse indicated to be unusual. The patient was able to explain that he felt “like he was in a bad dream and unable to wake up.” He denied visual or auditory hallucinations. His head was atraumatic and normocephalic. Cranial nerves I-XII were evaluated by neurological examination and found to be intact, with normal function in terms of visual acuity, extraocular movements, facial sensation and strength, hearing, gag reflex, and tongue movements. Distal motor and sensory functions were unremarkable in all extremities. Head CT without contrast indicated normal findings. Complete blood count, complete metabolic panel, and overall typical blood evaluations were unremarkable, with the exception of slightly elevated liver function enzymes expected due to of NAFLD and trace amounts of THC. Urinalysis toxicology was not performed due to technical laboratory challenges unique to urine specimens. The patient was diagnosed with “use of cannabinoid edibles” and was discharged in care of his spouse with guidance to follow-up with outpatient psychiatry and internal medicine.

Two days post-ED discharge, the patient’s spouse became concerned that the patient’s presentation was worsening. At home, the patient’s speech was subdued and increasingly delayed. He was sleeping all day and unable to leave bed. In the evening of the second day post-discharge, the patient began to report an encounter with God and spoke in Bible verses. He expressed that he felt pulled in between the earth and the divine. He repeated verses from scripture and sang religious hymns. In conversation with the patient’s spouse, the patient made the comment “the father commands you” likely in reference to God the Father as part of the Holy Trinity in Christianity. He was noticeably agitated and upset. Notably, the patient has an extensive academic background in religion and identified as religious. The patient’s spouse was clearly very disturbed and distressed due to the patient’s presentation and brought the patient back for a secondary evaluation. The patient was evaluated by psychiatry and diagnosed with CIP.

Following a psychiatric consultation, the patient was prescribed 10 mg aripiprazole once daily for two weeks and then 5 mg for an additional two weeks. It was also recommended that the dosage be reduced to 2.5 mg for the following two weeks but the patient reported no continued symptoms and declined additional follow-up and treatment. The patient was recommended to follow up with outpatient psychiatry via telemedicine. The patient and his spouse were educated about warning signs of an affective or psychotic relapse (e.g., changes in sleep cycle, social withdrawal, cognitive difficulties), with a recommendation to continue outpatient therapy to reduce the likelihood of a subsequent psychotic episode.

Once home, the patient continued to experience excessive daytime sleepiness (EDS). After two days of treatment, the patient reported that the visual hallucinations had stopped. After four days of treatment, EDS has declined, and the patient’s speech, behavior, and overall presentation markedly improved. One week post-treatment, the patient reported complete normalcy with no apparent lasting symptomatology.

## Discussion

The precise mechanisms underlying CIP are not yet fully understood, but several theories have been proposed to explain its development. One prominent theory regarding traditional cannabis suggests that the interaction between the psychoactive compound Δ9-THC and the endocannabinoid system in the brain contributes to the onset of psychosis. The endocannabinoid system plays a crucial role in regulating various cognitive and emotional processes, and the disruption of its normal functioning by Δ9-THC may lead to psychotic symptoms [12]. Additionally, the genetic vulnerability of certain individuals to psychosis may interact with cannabis use, further increasing the risk of developing CIP. Another hypothesis proposes that the imbalance of neurotransmitters, such as dopamine and glutamate, may be involved in the pathophysiology of CIP. Cannabis use has been shown to affect dopaminergic and glutamatergic neurotransmission, which can impact neural circuitry associated with psychosis [13]. Alterations in these neurotransmitter systems may contribute to the emergence of psychotic symptoms in susceptible individuals.

One of the noteworthy aspects of this case is the delayed onset of psychotic symptoms following the initial cannabis intoxication. It has been documented that cases of CIP can occur with edible use and vary significantly in symptom duration [14]. However, this case differs in that symptoms developed days after ingesting cannabis. The patient began to display atypical behaviors, such as forgetting personal belongings and missing work and school assignments. While cannabis withdrawal syndrome is commonly observed in heavy cannabis users, this patient's history revealed limited cannabis use with only a handful of instances. However, it is worth noting that the dose consumed was relatively high and may have played a role in triggering psychotic withdrawal symptoms, which typically are associated with higher dosages. Withdrawal symptoms, such as irritability, anxiety, insomnia, and mood disturbances, can also manifest within days after discontinuing cannabis use. The emergence or exacerbation of psychosis during cannabis withdrawal has been reported, underscoring the importance of considering this possibility even in individuals with less frequent cannabis use.

Another significant feature of this case is the evolution of symptoms toward religious-themed psychosis. The patient’s increasing confusion, altered speech, and preoccupation with religious concepts raised the possibility of a spiritual or existential crisis triggered by CIP. Given the patient’s extensive academic background in religion and his self-identification as religious, it is plausible that these factors played a role in the content of his delusions and hallucinations. The emergence of religious themes in psychosis highlights the importance of considering the patient's individual cultural and personal context when assessing and treating psychotic symptoms.

The diagnosis of CIP in this case was not immediately evident during the initial ED evaluation. The patient’s symptoms were initially attributed to cannabis intoxication, and the presence of trace amounts of THC in his system supported this assumption. However, the worsening of symptoms after discharge and the development of psychosis prompted a reassessment of the diagnosis. It is essential to recognize that CIP, as well as other forms of psychosis, can still present with negative symptoms such as social withdrawal, flattened affect, and cognitive impairment, which were seen in the patient's initial presentation. The delay in identifying CIP, in this case, raises awareness about the need for comprehensive evaluations and follow-up assessments to ensure accurate diagnosis and appropriate management of psychiatric emergencies in the emergency department.

The initiation of antipsychotic treatment, specifically aripiprazole, was shown to be effective in managing the patient's symptoms. Within four days of starting treatment, there was a marked improvement in the patient’s speech, behavior, and overall presentation. While aripiprazole can have side effects including sedation, akathisia, extrapyramidal symptoms, weight gain, and metabolic disturbances, atypical antipsychotics such as aripiprazole are generally associated with a lower risk of extrapyramidal symptoms and tardive dyskinesia compared to first-generation antipsychotics [15]. Aripiprazole has previously shown efficacy and greater tolerability in the treatment of cannabis-related disorders, making it an appropriate choice for this patient [16] While there is a limited amount of studies to assess the efficacy of pharmacological interventions in CIP, leaving differences in antipsychotics unclear, some case studies have shown that olanzapine with haloperidol and lurasidone may be appropriate as well [11]. Adjunctive non-pharmacological interventions, such as supportive care, therapy, and psychoeducation, should also be incorporated into the treatment plan to enhance recovery and reduce the risk of recurrence.

Increased initiation of antipsychotic medication in the ED could possibly benefit patients presenting with severe and debilitating symptoms, possibly allowing them relief in the time it takes to arrange care with outpatient psychiatry as well as reducing the risk of later related agitation, as was seen in this case. However, there can be hesitancy among physicians to initiate psychiatric care in the ED, especially atypical antipsychotics, possibly leading to delays and prolonged debilitation for patients. This reluctance may stem from the concerns around side effects. It is also possible there may be challenges associated with finding time to manage psychiatric conditions in the fast-paced and resource-limited ED environment, causing physicians to defer psychiatric treatment to other services such as specialty consultations, outpatient follow-up, or transfer to a different unit.

To address this issue and improve outcomes for patients, it is crucial to increase awareness and training among emergency physicians regarding the management of psychiatric and cannabis-related emergencies. By equipping them with the necessary skills and knowledge, physicians can feel more confident in the management and treatment of behavioral emergencies in the ED. Additionally, fostering collaboration between emergency medicine and psychiatric teams is essential. This collaboration can streamline communication channels, establish protocols for timely psychiatric consultations, and ensure that patients receive the necessary psychiatric follow-up care without undue delays. By bridging the gap between acute management in the ED and ongoing psychiatric treatment, early intervention can alleviate distress, improve patient functioning, and prevent prolonged periods of debilitation.

## Conclusions

This case report presents a distinctive occurrence of CIP in a 22-year-old male, who exhibited initial symptoms of derealization and altered mental status post-consumption of a cannabinoid-infused gummy worm. Despite the resolution of acute effects within a day, the patient experienced a progressive intensification of psychotic symptoms, culminating in religious-themed psychosis. This case underscores the diagnostic complexities associated with CIP, especially when presenting with negative symptoms akin to primary psychiatric disorders or when initial symptoms are misattributed to cannabis intoxication alone, emphasizing the need for prompt and comprehensive assessment and care in such presentations.
